# Supplementary material for: Microbial contamination of spittoons and germicidal effect of irradiation with krypton chloride excimer lamps (Far UV-C 222 nm)
Source: PLoS One. 2024 Aug 7;19(8):e0308404. doi: 10.1371/journal.pone.0308404 (PMC11305547; doi:10.1371/journal.pone.0308404)
Supplement: Attachment — Submitted filename: Response_to_Reviewers.docx [file pone.0308404.s001.docx]

March 18, 2024.

**Dr. Charles Odilichukwu R. Okpala**

***PLOS ONE***

**Dear Dr. Okpala,**

We wish to re-submit the manuscript titled “Microbial contamination of spittoons and germicidal effect of irradiation with Krypton Chloride excimer lamps (Far UV-C 222 nm).” The manuscript ID is PONE-D-23-38195.

We thank you and the reviewers for your thoughtful suggestions and insights. The manuscript has benefited from these insightful suggestions. I look forward to working with you and the reviewers to move this manuscript closer to publication in PLOS ONE.

The manuscript has been rechecked and the necessary changes have been made in accordance with the reviewers’ suggestions. The responses to all comments have been prepared and provided below. The changes in the manuscript are in red text.

Thank you for your consideration. I look forward to hearing from you.

Sincerely,

Hiroaki Tanimoto

Address: 1-5-17 Otenae, Chuo-ku, Osaka 540-0008, Japan

Email: tanimoto@cc.osaka-dent.ac.jp

**Major concern**

**Comment 1:** The main issue is that each method is included in a single paragraph. It ought to be divided into multiple sections. Each technique must to have a unique the title. Every protocol should be written in a single, concise sentence. Every protocol should be thoroughly written

**Response:** Thank you for your insightful feedback. We have accordingly divided the study Methods into three subsections: “Biological contamination of spittoon” (lines 84–126), “Bacterial susceptibility to Far UV-C *in vitro*” (lines 132–147), and “Disinfection efficacy of Far UV-C irradiation on spittoon” (lines 148–170). Furthermore, within the “Biological contamination of spittoon” subsection, we have categorized the use of multiple assessment techniques under the headings “Swab sampling” (lines 85–93), “DNA extraction, library construction, and high-throughput sequencing” (lines 94–113), “Sequence data processing” (lines 114–126), and “Virus detection” (lines 127–131). Additionally, we have noted in the “Data Availability” statement (lines 263–268) that the data are currently being registered in a database. Moreover, in the Results section, we organized the data into distinct subsections under the headings “Biological contamination of spittoon” (lines 172–185), “Bacterial susceptibility to Far UV-C in vitro” (lines 192–196), and “Disinfection efficacy of Far UV-C irradiation on spittoon” (lines 203–216).

**Comment 2:** Did you create a growth curve for line 105, "First, each strain was grown to the mid-logarithmic growth phase"? How did you find out each strain's mid-logarithmic development phase?

**Response:** We appreciate your thorough confirmation. Yes, we obtained the growth curves of each bacterium by measuring the turbidity of the medium over time. This allowed us to determine the mid-log phase of each bacterium. We have described this in the revised Methods in lines 137–142.

**Comment 3:** The 16s rRNA sequencing technique is absent. This is a significant experiment; could you further elaborate on the 16s rRNA sequencing methodology?

**Response:** We acknowledge the oversight and understand the significance of including a detailed description of this methodology in our study. Hence, we have carefully revised the Methods section of our manuscript to incorporate a comprehensive elaboration on the 16S rRNA sequencing methodology. Specifically, we detailed the 16S rRNA sequencing process, including the primer sequences used, the PCR conditions, the sequencing platform, and the bioinformatics analysis pipeline (lines 94–113).

**Comment 4:** The manuscript needs to include the steps for phylogenetic diversity, OTU grouping and taxonomical assignment, trimmed sequence, and data sequence filtering in QIIME 2.

**Response:** We sincerely appreciate your thorough review and valuable comments on our manuscript. Your insights have significantly contributed to enhancing the quality and depth of our study. We have revised the Methods section to include comprehensive details on the conditions used for trimming, the formation of Amplicon Sequence Variants (ASVs) using DADA2, ASV grouping and taxonomical assignment, and sequence filtering. These additions were aimed to provide clarity on the bioinformatics pipeline and analytical processes employed in our study. Regarding the phylogenetic diversity analysis, we would like to clarify that owing to the limited number of samples analyzed in our study (n=3), we were unable to perform this analysis. A larger dataset would be required to conduct a meaningful phylogenetic diversity analysis, and thus, we have focused our efforts on providing robust data and analysis for the aspects directly relevant to our study’s objectives. These updates and clarifications can be found in lines 114–126 in the revised Methods section of our manuscript.

**Comment 5:** How did you determine that the change is significant? The paper has to include alpha and beta diversity analysis along with figures. How many germs are extracted from the sample and how many are common? There should be a Venn diagram.

**Response:** We primarily focused on evaluating the bactericidal effects of Far UV-C light on oral streptococci and bacteria present on sink surfaces. The main goal was to assess the potential of Far UV-C as a disinfection strategy, rather than conducting an exhaustive microbial diversity analysis. Although we did investigate the bacterial composition present on sink surfaces, this was performed with the intent to broadly understand the types of bacterial communities affected by Far UV-C treatment. Notably, our study analyzed a limited number of samples (n=3), which inherently restricted our ability to perform comprehensive statistical analyses, including alpha and beta diversity analyses. The sample size was chosen based on the study objective of providing preliminary evidence of Far UV-C’s bactericidal effects under the conditions tested, rather than to explore the microbial diversity in depth.

Regarding the extraction of germs from the samples and their commonality, our analysis was designed to identify the presence of bacterial groups rather than quantify their abundance or perform comparative analyses across a large dataset. Consequently, we did not include a Venn diagram or similar figures that would imply a statistical comparison of microbial communities across multiple samples. We understand the importance of microbial diversity analysis in many studies and agree that such analyses, including the use of Venn diagrams to illustrate common and unique taxa among samples, can provide valuable insights. However, given the focused scope of our research and the limited number of samples, we believe that extensive diversity analyses were beyond the objectives of this preliminary study. We hope this response clarifies the rationale behind the methodologies employed and the scope of our analysis.

**Minor Comments**

**- Please add reference to line 43-44, 50- 51**

Response: We have added the appropriate citations (**line 44-45**: [1] Kohn WG, Collins AS, Cleveland JL, Harte JA, Eklund KJ, Malvitz DM, et al. Guidelines for infection control in dental health-care settings--2003. MMWR Recomm Rep. 2003;52(RR-17): 1-61.**, line 51**: [4] Alvarenga MOP, Veloso SRM, Bezerra ALCA, Trindade BP, Gomes ASL, Monteiro GQM. COVID-19 outbreak: Should dental and medical practices consider uv-c technology to enhance disinfection on surfaces? - A systematic review. J Photochem Photobiol. 2022;9: 100096.doi:10.1016/j.jpap.2021.100096.).

**- Italicize the bacterial name as in line 139 “Pseudomonas,” Pls check the whole manuscript.**

Response: Thank you for your feedback. We have made the corrections and italicized all scientific names. Additionally, we have conducted a thorough review of the entire manuscript regarding this matter.

**- Line 84, change “was attempted” to “ was conducted”**

Response: Thank you for your feedback. This was revised accordingly (lines 87)

**- Line 93, what is the size of amplified fragment? Add primers sequences used?**

Response: Thank you for your insightful comment and for pointing out the missing details regarding the size of the amplified fragment and the primer sequences used in our study. We have accordingly updated the Methods section of our manuscript to include the specific size of the amplified fragment as well as the sequences of the primers used. This additional information should provide a clearer understanding of our experimental approach and facilitate the reproducibility of our study. Please refer to lines 94–113 in the revised Methods section for the detailed information.

**- Line 106, “and suspended in PBS? Is not clear, GAM broth or PBS?. Write the whole protocol in details.**

Response: Thank you for your helpful suggestions to aid the understanding of our readers. We have clarified that it is a dilution with PBS and consolidated it into one paragraph (lines 132–147).

**- Lines 107–109 This procedure is quite brief. "How did you perform the irradiation? Could you perhaps elaborate on the protocol in a separate paragraph?**

Response: Thank you for your valuable feedback. We appreciate your pertinent suggestion and have included information regarding the previously omitted irradiation dosage (lines 142–145).

**- Lines 109–110 The CFU method should be described in detail as well.**

Response: Thank you for your feedback. We have included the culture conditions in lines 146–147.

**- Line 115 “Our results suggest that 222 nm Far UV-C irradiation may inhibit the pathway of bacterial transmission by droplets from sink surfaces.” Should be moved to result.**

Response: Thank you for your insightful opinion regarding our suggestion. However, because our study did not directly evaluate the inhibition of transmission by droplets, we would like to retain this aspect for discussion.

**- Line 130, “number of bacteria” Do you mean CFU? Why did you count 300 colonies when there were actually more?**

Response: Thank you for your review. “Number of bacteria” refers to CFU. Based on your feedback, we have corrected the term to CFU (line 168-169). Additionally, we considered samples with more than 300 CFU to be outliers. Moreover, given an adequate sample size, we concluded that the trend of bacterial reduction due to Far UV-C irradiation was not significantly affected.

**- Line 141-142 “Ten of these species were present in three samples with more than 0.5% in common” Is it among the 15 sample or among the whole species numbers? You need to explain or justify why it precent in very low percentage.**

Response: Thank you for your constructive feedback and for highlighting areas of our manuscript that required clarification. We appreciate the opportunity to improve our manuscript and make our findings more accessible to our readers.

In response to your comment regarding the specificity of the statement in lines 141–142, we have revised the text to clearly indicate that the reference to the ten species being present in more than 0.5% in common pertains to the top 15 species identified in our study, not the total number of species across all samples. This revision aimed to eliminate any ambiguity and make it easier for readers to understand the context of our analysis. Please refer to the revised text in the Results section (lines 178–183) for the updated information.

Additionally, we have added further explanation to address why these species are present in relatively low percentages. We have included information on the detection of various bacteria originating from the oral cavity and environmental sources, particularly water systems, which contributed to the observed diversity in bacterial composition among the three spittoons analyzed. This addition is intended to provide insight into the variability of the microbiota composition and the factors influencing the presence of different bacterial species at varying abundances. The details of this explanation can be found in the Results section (lines 178–183). We believe these revisions and additions will enhance the clarity and comprehensiveness of our manuscript, and we are grateful for your suggestions that guided these improvements.

**- Enter the complete names of the SD, SE, and IC below the Table 1 as short notes. Include the "CFU" in the table itself as well.**

Response: Thank you for your feedback. We have included these descriptions in lines 228–229 as an explanation for Table 1.

**-A native English speaker should review the paper. Please review the entire document.**

Response: Thank you for your feedback. The paper has been proofread and edited by a native English speaker, and we have acknowledged this in the Acknowledgments section (lines 256-257).

We **have made corrections to the following three points:**

1. There was an error in the designation of affiliation (lines 14–16).
2. The English name of the kit needed correction (lines 91).
3. There was a typo in the mean value of After Irradiation for “Mitis Salivarius medium (n=60)” in Table 1 (lines 215, and 227–228).
