# Supplementary material for: Microbial contamination of spittoons and germicidal effect of irradiation with krypton chloride excimer lamps (Far UV-C 222 nm)
Source: PLoS One. 2024 Aug 7;19(8):e0308404. doi: 10.1371/journal.pone.0308404 (PMC11305547; doi:10.1371/journal.pone.0308404)
Supplement: Attachment — Submitted filename: Response to Reviewers_ver.3.docx [file pone.0308404.s002.docx]

**Dear Dr. Charles Odilichukwu R. Okpala**

**Academic Editor**

**PLOS ONE**

Thank you for giving me the opportunity to submit a revised draft of my manuscript titled “Microbial contamination of spittoons and germicidal effect of irradiation with Krypton Chloride excimer lamps (Far UV-C 222 nm)” to *PLOS ONE*. We appreciate the time and effort that you have dedicated to providing your valuable feedback on my manuscript.

I am/We are grateful to your insightful comments on my paper. We have been able to incorporate changes to reflect some of the suggestions provided. We have highlighted the changes within the manuscript.

**Editor Comments**

**Comment** **a)** Kindly include in your introduction about the global ramifications of microbial contamination in clinical settings - mortality, etc, bring in published data showing different continents, narrow down to Asia, then one in Japan. (this should be your paragraph 1). Make sure to contextualize the paragraph 1 to the subject of this work.

**Response:** Thank you for pointing that out. The focus of our study was not on survival rates or mortality rates in hospital-acquired infections, but on reducing biological contamination in dental treatment booths during dental treatment, so we were unable to obtain data on research from this perspective. On the other hand, there were studies on the mortality rates of hospital-acquired infections worldwide by pathogenic bacteria, but these were not directly linked to the target of this study, which was to reduce biological contamination.

**Comment b)** In materials and methods, please separate out the data analysis aspects and create the last subsection "statistical analysis", and have all of them there.

**Response:** Thank you for your suggestions to make the clearer. We have created a subsection "Statistical Analysis" in "Materials and Methods" to summarize the data. [Line 170 - 176]

**Comment c)** Your discussion needs additional depth. Please, provide deeper explanation of the results, not just about 'what', but more about 'how' and 'why?' . Please also, kindly make sure you include (Refer to Table 1) (Refer to Figure 2) (Refer to Figure 3) (Refer to Figure 4) in all the places where data of each has been discussed. So, all the Tables/Figures in the results, must be captured in the discussion

**Response:** Thank you for your comments that deepened the discussion appropriately. I have added missing figures and tables [Line 245, 247, 252] and incorporated all figures and tables in the results into the discussion. Also, to clarify the relationship between existing reports and our results [why], I have clarified the amount of Far UV-C irradiation and the inactivation amount of Pseudomonas aeruginosa in the existing results. [Line 242-243] On the other hand, I have deleted the phrase "which were only rinsed with flowing water," which is not directly related to the comparison of irradiation and non-irradiation [Line 249].
